# Vibration Analysis of Carbon Fiber-Graphene-Reinforced Hybrid Polymer Composites Using Finite Element Techniques

**DOI:** 10.3390/ma13194225

**Published:** 2020-09-23

**Authors:** Stelios K. Georgantzinos, Georgios I. Giannopoulos, Stylianos I. Markolefas

**Affiliations:** 1 Laboratory for Advanced Structures and Smart Systems, General Department, National and Kapodistrian University of Athens, 34400 Psachna, Greece; 2 Department of Mechanical Engineering, University of Peloponnese, 1 Megalou Alexandrou Street, 26334 Patras, Greece; ggiannopoulos@uop.gr; 3 General Department, National and Kapodistrian University of Athens, 34400 Psachna, Greece; stelmarkol@uoa.gr

**Keywords:** composite structures, graphene, carbon fiber, hybrid matrix, vibrations, finite element analysis

## Abstract

In this study, a computational procedure for the investigation of the vibration behavior of laminated composite structures, including graphene inclusions in the matrix, is developed. Concerning the size-dependent behavior of graphene, its mechanical properties are derived using nanoscopic empiric equations. Using the appropriate Halpin-Tsai models, the equivalent elastic constants of the graphene reinforced matrix are obtained. Then, the orthotropic mechanical properties of a composite lamina of carbon fibers and hybrid matrix can be evaluated. Considering a specific stacking sequence and various geometric configurations, carbon fiber-graphene-reinforced hybrid composite plates are modeled using conventional finite element techniques. Applying simply support or clamped boundary conditions, the vibrational behavior of the composite structures are finally extracted. Specifically, the modes of vibration for every configuration are derived, as well as the effect of graphene inclusions in the natural frequencies, is calculated. The higher the volume fraction of graphene in the matrix, the higher the natural frequency for every mode. Comparisons with other methods, where it is possible, are performed for the validation of the proposed method.

## 1. Introduction

Composites are made up of two or more constituent materials that, when combined, generate a material with physical and chemical characteristics different from the individual components. Composite structures have presented outstanding advances in the last decades. The recent applications of fiber-reinforced composite structures to marine [1], civil [2], aerospace [3] and other industries [4,5,6] have already been reviewed in the open literature. Fast progress in manufacturing has resulted to the necessity for the improvement of composites in terms of strength, stiffness, density, and lower cost.

Graphene is a carbon allotrope, the thinnest known material, i.e., one carbon atom thick, which additionally is exceptionally strong—almost 200 times stronger than steel [7,8,9]. Furthermore, graphene presents outstanding electric [10,11], heat [12] and optical [13] properties. The excellent properties of graphene make it an excellent candidate as reinforcing material in composites. Consequently, many studies can be found in the literature investigating experimentally [14,15,16,17], computationally [18,19,20], or analytically [21,22] the effect of graphene in nanocomposites. The superb behavior of graphene nanocomposites has driven the research and technology for finding advanced applications. Chang and Wu [23] have reported recent energy-related development of graphene nanocomposites in solar energy conversion, such as photovoltaic and photoelectrochemical devices and artificial photosynthesis, as well as electrochemical energy devices and improvements in environmental applications of functionalized graphene nanocomposites for the detection and removal of heavy metal ions, organic pollutants, gas and bacteria. Kumar et al. [24] have systematically examined the recent progress in illustrating the complexities of mechanical and thermal behavior of graphene- and modified graphene-based polymer nanocomposites. They also reported the potential applications, existing challenges, and potential perspectives regarding the multi-scale capabilities and promising advancements of the graphene family-based nanocomposites materials.

The fiber and matrix-dominant behavior of fiber-based polymer composites are crucial in many mechanical, aerospace, or structural applications. Pre-mixing the polymer matrix with nanoparticles could enhance the through-thickness or matrix-dominant properties. Rafiee et al. [25] employed several graphene-based nanomaterials, including graphene oxide and its thermally reduced version (rGO), graphene nanoplatelets, and multi-walled carbon nanotubes to modify the epoxy matrix and the surface of glass fibers. Unmodified and modified epoxy and fibers were used for fabricating multiscale glass fiber-reinforced composites, following the vacuum-assisted resin transfer molding process. The composites obtained combined improvements in both the fiber and matrix-dominant properties, resulting in superior composites. Kostagiannakopoulou et al. [26] developed a new class of carbon fiber-reinforced polymers with a nano-modified matrix, based on graphene nano-species. Their results have shown that nano-doped composites present a significant improvement of the interlaminar strain energy release rate of the order of 50% for the case of graphene nano-platelets. Taş and Soykok [27] have determined engineering constants of carbon nanotube based unidirectional carbon fiber-reinforced composite lamina theoretically with two different approaches. They have modeled using the finite element method and analyzed the behavior of a specific stacking sequence and configuration of a composite plate under static loadings. Their results have shown a considerable improvement of the plate stiffness due to the presence of nanotubes.

Although some works have already been done on vibrations of composite structures reinforced by nanoparticles, there are only a few works, which focused on vibration behavior of carbon fiber-based laminate composites with pure graphene inclusions. Hulun et al. [28] have investigated free vibration of graphene nanoplatelet (GPL) reinforced laminated composite quadrilateral plates using the element-free IMLS-Ritz method. They concluded that increasing GPLs weight fraction can increase the natural frequency of quadrilateral plate. Shen et al. have investigated nonlinear vibration of functionally graded graphene-reinforced composite laminated plates [29], functionally graded graphene-reinforced composite laminated cylindrical panels resting on elastic foundations [30], and functionally graded graphene-reinforced composite laminated cylindrical shells [31] in thermal environments. Wang et al. [32] have proposed a two-dimensional elasticity model of laminated graphene-reinforced composite (GRC) beams, where the graphene disperses uniformly in each layer, but the graphene volume fraction may vary from layer to layer. It is found that the laminated GRC beam with graphene distribution pattern X has the least deflection and highest fundamental frequency at extreme length-to-thickness ratios, but it has the highest deflection and least fundamental frequency at a very low length-to-thickness ratio due to its reduced transverse shear stiffness.

In the present paper, a computational approach is proposed for the estimation of vibration characteristics of carbon fiber laminate composite plates with graphene inclusions. The method firstly computes the size-dependent mechanical properties of graphene used in the composite. In the second phase, the orthotopic properties of the hybrid matrix are calculated adopting Halpin-Tsai models for specific volume fractions of graphene in the matrix. Knowing the elastic constants of hybrid matrix, the elastic mechanical properties of the composite lamina can be evaluated. In the next step, finite element models are developed for various configurations of laminated composite plates using conventional shell elements. Applying different boundary conditions and solving the free vibration problem, modes of vibration and corresponding natural frequencies are finally extracted. The effect of graphene volume fraction on vibration characteristics of various composite plates is determined. To the authors’ best knowledge, it is the first time where this holistic and relatively simple approach (four-stage technique), taking into account the size-dependent behavior of graphene inclusions, is proposed for the vibration analysis of graphene-carbon fiber-reinforced composite structures.

## 2. Computational Approach

### 2.1. Problem Definition

**Figure 1:**
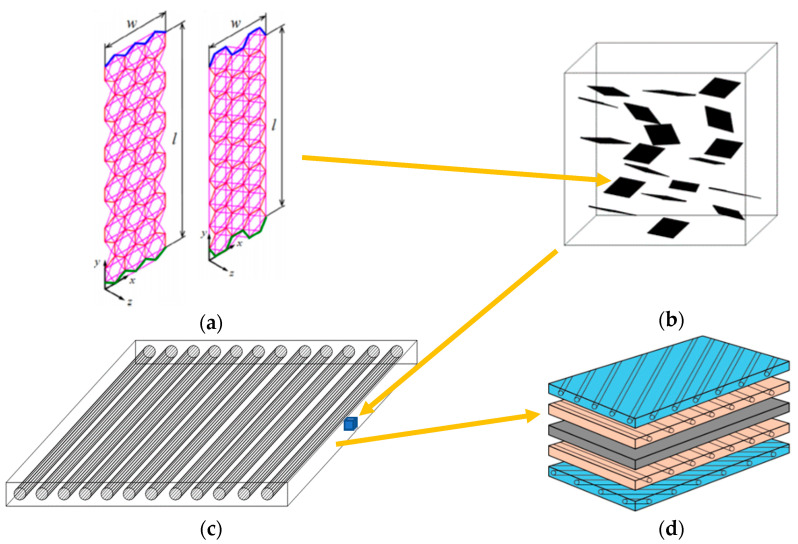
Modeling procedure (four-stage technique) of a laminated composite plate with graphene inclusions (**a**) finite element model of graphene in nanoscale; (**b**) homogenized model of hybrid matrix; (**c**) composite lamina with hybrid matrix; (**d**) composite plate with specific stacking sequence.

Figure 1 depicts the composite plate considered to be analyzed. In Figure 1a, the finite element model of the graphene in nanoscale is illustrated. The information about the size-dependent mechanical performance of graphene derived from nanoscale is used for the determination of the elastic constants of hybrid matrix (Figure 1b) using Halpin-Tsai homogenization model. The elastic constants of the hybrid matrix are utilized in order to compute the orthotropic mechanical properties of the unidirectional composite lamina (Figure 1c). Then, the finite element modeling of a composite plate considering a specific stacking sequence (Figure 1d) can be performed for its vibration analysis.

### 2.2. Graphene Mechanical Properties in Nanoscale

The elastic mechanical behaviour of different-sized graphene can be numerically examined and predicted using a spring-based finite element approach [7,33]. According to those approaches, three-dimensional, two-nodded, spring-based, finite elements of three degrees of freedom per node are combined in order to model effectively the interatomic interactions presented inside the graphene. The calculated variations of mechanical elastic constants have been approached by appropriate size-dependent non-linear functions of two independent variables, i.e., length and width for graphene, in order to express the analytical rules governing the elastic behaviour. The numerical results have been already validated through comparisons with corresponding data given in the open literature.

The Young’s modulus in TPa of the graphene can be predicted using the following rational function [33]

(1) $$E_{gr}\left(w_{gr},l_{gr}\right)=\frac{P_{0}+a_{01}w_{gr}+b_{01}l_{gr}+b_{02}l_{gr}{}^{2}+c_{02}w_{gr}l_{gr}}{1+a_{1}w_{gr}+b_{1}l_{gr}+a_{2}w_{gr}{}^{2}+b_{2}l_{gr}{}^{2}+c_{2}w_{gr}l_{gr}},0<w_{gr},l_{gr}\leq 10\;\mathrm{nm}$$

where $P_{0},a_{01},b_{01},b_{02},c_{02},\;a_{1},b_{1},a_{2},b_{2},\mathrm{and}\;c_{2}\;$ are constants, which are determined by non-linear fitting, while $w_{gr}$ and $l_{gr}$ are the graphene width and length, respectively. The values of the constants of Equation (1), concerning both the two chirality directions of graphene are presented in Table 1. The accuracy of this equation compared to the finite element predictions and expressed by the coefficient of determination is calculated to be over than 99.5%.

### 2.3. Graphene/Polymer Matrix Physical Properties

The three-dimensional (3D) Halpin-Tsai model for randomly oriented discontinuous rectangular reinforcements is utilized to compute the Young’s modulus of the hybrid graphene/polymer matrix. According to this model, the Young’s modulus of the graphene/polymer matrix can be predicted by the following equation [34]

(2) $$E_{m-gr}=\frac{1}{5}E_{L}+\frac{4}{5}E_{T}$$

where

(3) $$E_{L}=\frac{1+\xi_{L}\eta_{L}V_{gr}}{1-\eta_{L}V_{gr}}E_{m}$$

(4) $$E_{T}=\frac{1+\xi_{T}\eta_{T}V_{gr}}{1-\eta_{T}V_{gr}}E_{m}$$

and

(5) $$\;\eta_{L}=\frac{\frac{E_{gr}}{E_{m}}\;-1}{\frac{E_{gr}}{E_{m}}+\xi_{L}},\;\eta_{T}=\frac{\frac{E_{gr}}{E_{m}}\;-1}{\frac{E_{gr}}{E_{m}}+\xi_{T}}$$

(6) $$\xi_{L}=2\frac{l_{gr}}{t_{gr}}+40V_{gr}{}_{}^{10},\;\xi_{T}=2\frac{w_{gr}}{t_{gr}}+40V_{gr}{}_{}^{10}$$

where $V_{gr}$ and $t_{gr}$ stand for volume fraction and thickness of graphene, respectively. Furthermore, $E_{m}$ is the Young’s modulus of the polymer matrix, while $\xi_{T}$, $\xi_{L}$, $\eta_{L}$, and $\eta_{T}$ are Halpin-Tsai parameters, depending on the geometry of the reinforcement.

Assuming that Poisson’s ratio $v_{m}$ for both pure polymer matrix and hybrid matrix are approximately similar, it is calculated that Poisson’s ratio of graphene/polymer matrix is

(7) $$v_{m-gr}=v_{m}$$

Consequently, the shear modulus of the hybrid matrix exhibiting quasi-isotropic behaviour may be evaluated from the next equation

(8) $$G_{m-gr}=\frac{E_{m-gr}}{2\left(v_{m-gr}+1\right)}$$

Considering the inertia effect for the dynamic problem, the equivalent mass density of the hybrid matrix can be determined using rule of mixtures as

(9) $$\rho_{m-gr}=V_{gr}\rho_{gr}+V_{m}\rho_{m}$$

where $\rho_{gr}$,$\;\rho_{m}$, and $V_{m}$ are the mass density of graphene, mass density of matrix, and the volume fraction of the matrix, respectively. It is also known that

(10) $$V_{gr}+V_{m}=1$$

### 2.4. Physical Properties of Composite Lamina

Considering a unidirectional lamina, its Young’s modulus in longitudinal direction, E_1_, and Poisson’s ratio, *υ*_12_ can be computed by employing the standard rule of mixture [35] as follows:

(11) $$E_{1}=E_{f11}\;V_{f}+E_{m-gr}V_{m-gr}$$

(12) $$\upsilon_{12}=\upsilon_{f}V_{f}+\upsilon_{m-gr}V_{m-gr}$$

where *E_f_*_11_, *V_f_*, *υ_f_* and *V_m-gr_* are Young’s modulus of fiber along in longitudinal direction, volume fraction of fiber, Poisson’s ratio of fiber and volume fraction of the hybrid matrix, respectively. The remaining orthotropic material properties, i.e., Young’s modulus in transverse direction, *E*_2_, Poisson’s ratio, *υ*_23_, and shear moduli, *G*_12_ and *G*_23_, can be computed by the next general Halpin-Tsai [36] expression

(13) $$\frac{P}{P_{m-gr}}=\frac{1+\xi \eta V_{f}}{1-\eta V_{f}}$$

where *P_m-gr_* are the related properties of hybrid matrix. Moreover, *η* is an experimental factor, which is computed by using the next equation,

(14) $$\eta =\;\frac{\frac{P_{f}}{P_{m-gr}}-1}{\frac{P_{f}}{P_{m-gr}}+\;\xi }$$

where *P_f_* are the related properties of fiber. Notice that *ξ* is a measure of reinforcement geometry, which affected by loading conditions. Here, *ξ* is chosen as 2 for computation of *E*_2_ and as 1 for computations of *υ*_23_, *G*_12_ and *G*_23_.

Considering inertia effects, the equivalent mass density of the composite structure can be determined using rule of mixtures as

(15) $$\rho_{c}=V_{f}\rho_{f}+V_{m-gr}\rho_{m-gr}$$

where $\rho_{f}$,$\;\rho_{m-gr}$ and $V_{m-gr}$ are the mass density of the fiber, mass density of the hybrid matrix, and volume fraction of the hybrid matrix, respectively. It is also known that

(16) $$V_{f}+V_{m-gr}=1$$

### 2.5. Finite Element Modelling of the Composite Plate

In the present study, the finite element method is used for conducting free vibration analysis of the composite plates with graphene inclusion. For performing the vibration analysis, an appropriate mesh must be developed in the problem domain. Considering that the composite plate has a side-to-thickness ratio higher than 10, shell elements are an effective option for meshing the model. Here, 4-node, quadrilateral, stress/displacement shell elements with reduced integration and a large-strain formulation are used [37].

According to the ASTM D7264 standard, *w* × *l* × *t*, with *w*, *l* > 10 *t* rectangular composite plates are modelled, where *w* is the width, *l* is the length, and *t* is the thickness of the plate. The plates consist of 8 unidirectional layers. The thickness and the stacking sequence of all plates under consideration are 2 mm and [0/+45/−45/90]*s*, respectively. The model has been tested for its convergence in terms of mesh density and a mesh with a size of 0.5 mm for each element is finally chosen.

The hybrid composite plates of these characteristics, as well as different carbon nanostructure inclusion types and volume fractions, have been successfully validated [34] for bending loading conditions. Since the method is validated, it can be expanded for the solution of the free vibration problem. The elemental matrices and displacements are written in the global coordinate system by applying the appropriate transformation matrices. After generating the global stiffness matrix (***K***) and the global mass matrix (***M***), assembled from the elemental matrices using conventional finite element procedures and considering the undamped free vibrations of the composite plate, the equation of motion can be assembled as

(17) $$M\ddot{d}+Kd=0$$

where ***d*** is the assembled displacement vector. By applying the boundary conditions on the composite plate, the eigenvalue problem can be solved using common finite element procedures, which reveal the natural frequencies and corresponding mode shapes of vibration.

## 3. Results and Discussion

In this work, polyester resin, graphene, and carbon fiber were selected as matrix material, nano inclusion and reinforcement, respectively. Physical properties of the components constituting the hybrid composite material are presented in Table 2. These properties are the basis for the estimation of the lamina physical properties presented in the next section.

### 3.1. Effect of Graphene on Composite Lamina Physical Properties

Given the physical properties of the components, the physical properties of graphene-added unidirectional carbon fiber-reinforced lamina are evaluated theoretically, based on the proposed method, and the outcome is provided in Table 3. Note that *E*_1_, *E*_2_, *G*_12_, *G*_23_, as well as *ρ_c_* increase as the volume fraction of graphene increases. Specifically, there is an 34.6%, 542%, 533%, and 686% increase in *E*_1_, *E*_2_, *G*_12_, and *G*_23_ with the addition of 50 vol.% graphene, respectively. Simultaneously, there is only 13.6% increase in the mass density of the lamina due to the presence of graphene since it is denser than polyester resin.

### 3.2. Vibrational Characteristics of the Composite Plate

**Figure 2:**
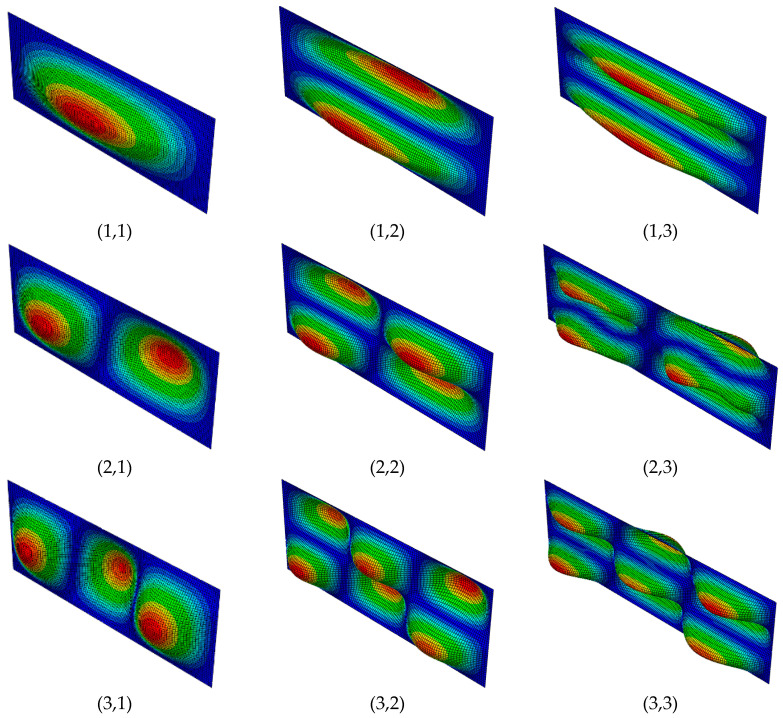
Mode shapes of vibration of a composite plate with *w* = 25 mm, *l* = 64 mm, and CCCC boundary conditions.

A numerical calculation of the natural frequencies and mode shapes of vibration can be carried out for a composite plate, according to the procedure described in the previous section for different boundary conditions. Here, we assume two types of supports, (a) the longitudinal and transversal edges of the rectangular plate are clamped, CCCC (clamped—clamped—clamped—clamped), and (b) the longitudinal and transversal edges of the plate are simply supported SSSS (simple—simple—simple—simple). Figure 2 depicts several basic bending mode shapes of vibration of the rectangular plate. The general nomenclature for the mode shapes, here, is (*a*,*b*), where *a* and *b* correspond to the number of ridges or valleys existing in the mode at longitudinal and transverse direction of the plate. Note that Figure 2 depicts the transverse modes of the CCCC case; however, the general shape (*a*,*b*) exists in the SSSS case as well. The difference in the shape is mainly on the boundaries, where the rotation of the degrees of freedom are free and fixed in SSSS and CCCC boundary condition, respectively. The structure of the first three (basic) modes of vibration depends on the plate geometric and stiffness characteristics. In general, the presence of graphene into the composite seems not to influence the shape of the vibration modes; however, it affects their sequence.

**Figure 3:**
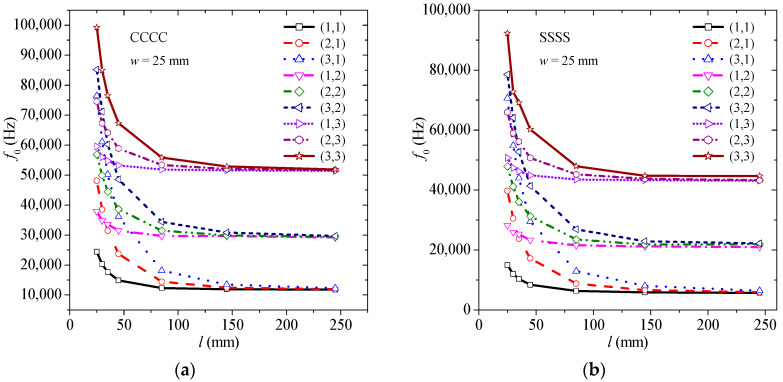
Natural frequency of the composite plate without the presence of graphene considering various configurations for (**a**) CCCC, and (**b**) SSSS boundary conditions.

Figure 3 depicts the natural frequency, *f*_0_, of the composite plate without the presence of graphene considering various configurations. Specifically, the width remains constant *w* = 25 mm, while the length is *l* = 25, 30, 35, 45, 85, 145, 245 mm. Figure 3a illustrates the natural frequency variation concerning CCCC boundary conditions. Figure 3b describes the natural frequency variation regarding SSSS boundary conditions. The natural frequencies of the CCCC case are greater than the corresponding ones of SSSS, as expected.

### 3.3. Effect of Graphene on Natural Frequancies Assuming under CCCC Boundary Conditions

**Figure 4:**
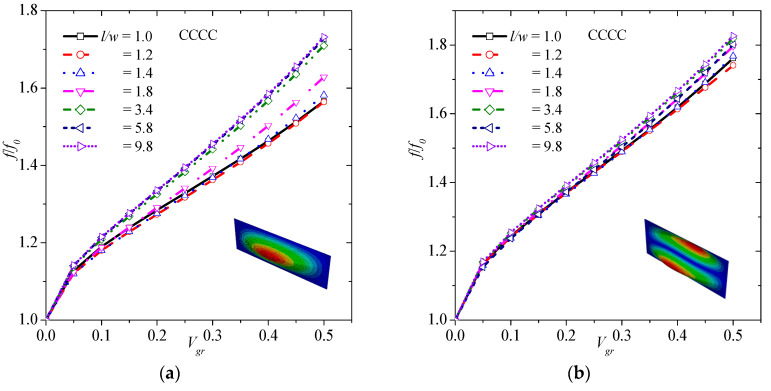

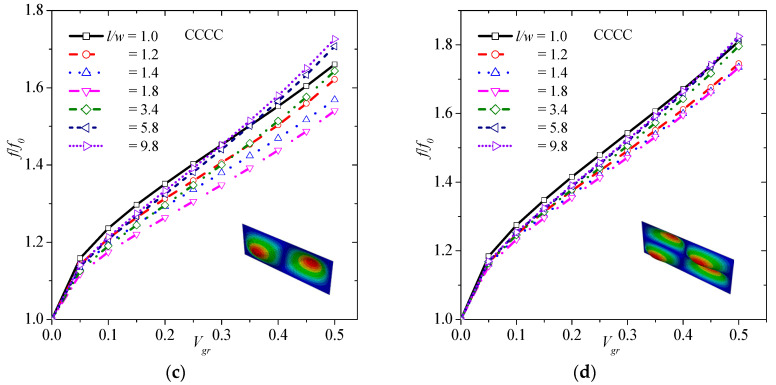
Normalized frequency versus volume fraction of graphene considering (**a**) (1,1), (**b**) (1,2), (**c**) (2,1), and (**d**) (2,2) vibration mode for the CCCC boundary condition.

In order to study the effects of graphene inclusion on vibration behavior of laminated composite plates under CCCC boundary conditions, various values of volume fraction of graphene are studied. Specifically, eleven different cases are considered, i.e., *V_gr_* = 0, 0.05, 0.10, 0.15, 0.20, 0.25, 0.30, 0.35, 0.40, 0.45, and 0.50. Figure 4 illustrates the effect of graphene volume fraction on (1,1) (Figure 4a), (1,2) (Figure 4b), (2,1) (Figure 4c), and (2,2) (Figure 4d) transverse vibrations. As observed, the higher the volume fraction of graphene, the higher the frequency of graphene. This observation is true for all the different transverse modes. Specifically, there is an up to 73.2%, 82.7%, 72.6%, and 82.4% increase in (1,1), (1,2), (2,1), and (2,2) mode shape, respectively, with the addition of 50 vol.% graphene.

**Figure 5:**
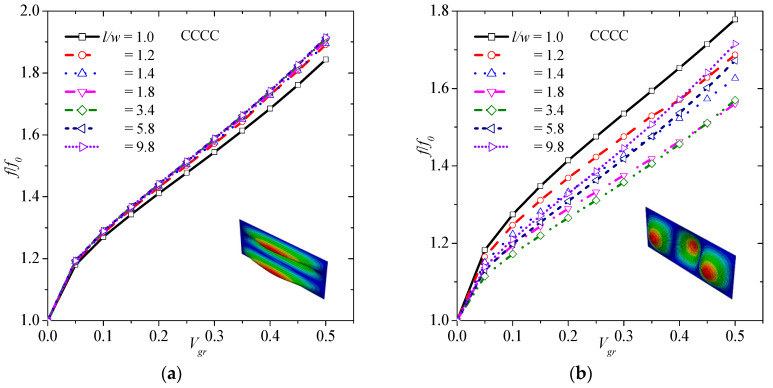

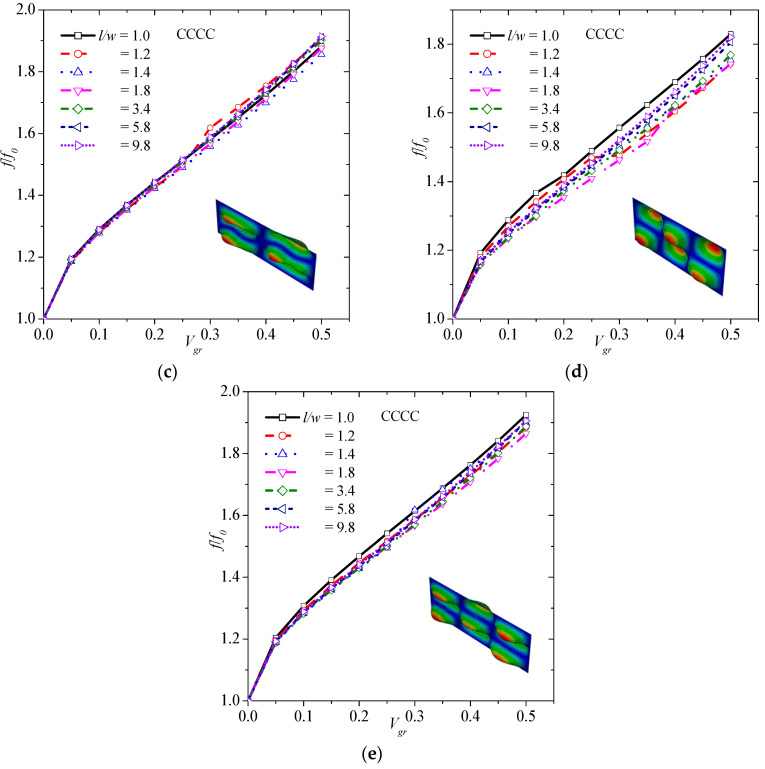
Normalized frequency versus volume fraction of graphene considering (**a**) (1,3), (**b**) (3,1), (**c**) (2,3), (**d**) (3,2), and (**e**) (3,3) vibration mode for CCCC boundary condition.

Figure 5 shows the effect of graphene volume fraction on (1,3) (Figure 5a), (3,1) (Figure 5b), (2,3) (Figure 5c), (3,2) (Figure 5d), and (3,3) (Figure 5e) transverse vibrations. As described previously, the higher the volume fraction of graphene, the higher the natural frequency of graphene. This observation is true for all different transverse modes. Specifically, there is an up to 91.5%, 71.5%, 91.4%, 82% and 90.6% increase in (1,3), (3,1), (2,3), (3,2), and (3,3), respectively, with the addition of 50 vol.% graphene. It is noted that the graphene increases slightly more the normalized frequency of higher order modes than basic ones.

### 3.4. Effect of Graphene on Natural Frequancies Assuming SSSS Boundary Conditions

In order to study the effects of graphene inclusion on vibration behavior of laminated composite plates under SSSS boundary conditions, various values of volume fraction of graphene are considered.

**Figure 6:**
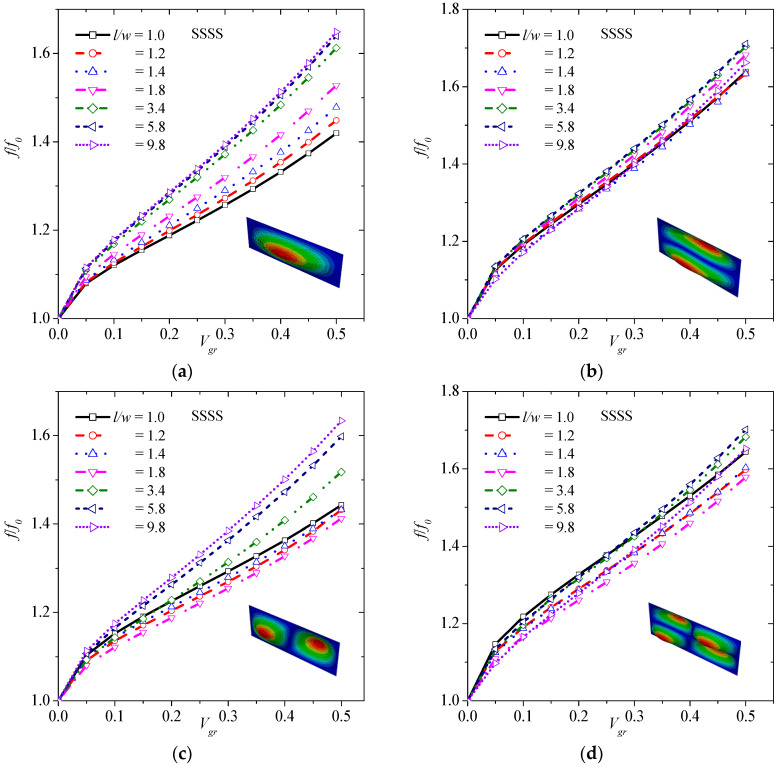
Normalized frequency versus volume fraction of graphene considering (**a**) (1,1), (**b**) (1,2), (**c**) (2,1), and (**d**) (2,2) vibration mode for SSSS boundary condition.

Once again, 11 different cases are considered, i.e., *V_gr_* = 0, 0.05, 0.10, 0.15, 0.20, 0.25, 0.30, 0.35, 0.40, 0.45, and 0.50. Figure 6 illustrates the effect of graphene volume fraction on (1,1) (Figure 6a), (1,2) (Figure 6b), (2,1) (Figure 6c), and (2,2) (Figure 6d) transverse vibrations. As observed, the higher the volume fraction of graphene, the higher the frequency of graphene for all different transverse modes. Specifically, there is an up to 64.9%, 60.9%, 63.4%, and 70.1% increase in (1,1), (1,2), (2,1), and (2,2) mode shape, respectively, with the addition of 50 vol.% graphene.

**Figure 7:**
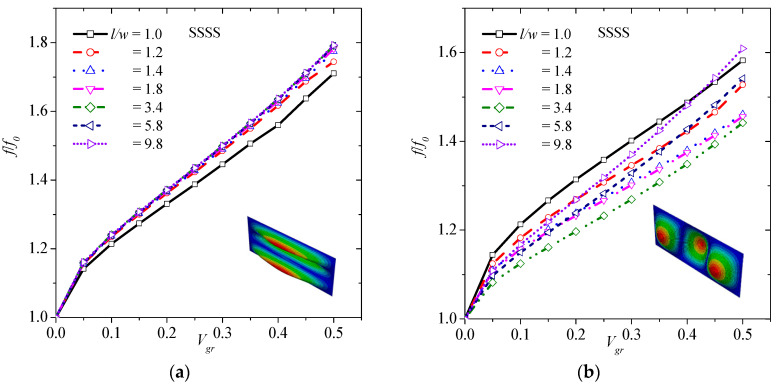

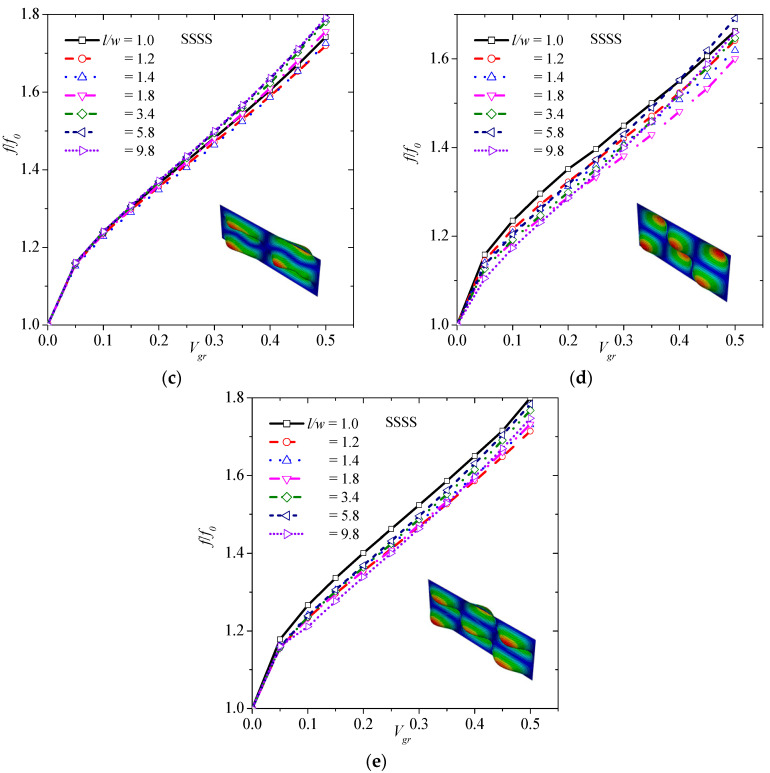
Normalized frequency versus volume fraction of graphene considering (**a**) (1,3), (**b**) (3,1), (**c**) (2,3), (**d**) (3,2), and (**e**) (3,3) vibration mode for the SSSS boundary condition.

Figure 7 shows the effect of graphene volume fraction on (1,3) (Figure 7a), (3,1) (Figure 7b), (2,3) (Figure 7c), (3,2) (Figure 7d), and (3,3) (Figure 7e) transverse vibrations. As previously, the higher the volume fraction of graphene, the higher the frequency of graphene. This observation is true for all the different transverse modes. Specifically, there is an 79.3%, 60.9%, 79.2%, 69.1% and 80% increase in (1,3), (3,1), (2,3), (3,2), and (3,3), respectively, with the addition of 50 vol.% graphene. As in SSSS case, the graphene seems to increase slightly more the normalized frequency of higher order modes than basic ones.

In the previous cases (SSSS and CCCC), the behavior of the normalized frequency of various vibration mode versus the graphene volume fraction has been depicted concerning different L/w ratio. It is observed that the slope of this curve changes with different L/w ratio, and the trend is also different for the different vibration mode. The effect is more obvious in lower order than higher order modes. This phenomenon may be explained by the variation of the stiffness and mass (density) characteristics of the structure due to the presence of graphene that differs from each other (Table 3). Nevertheless, as is well known, mass and stiffness ratio directly affect the vibration frequency.

### 3.5. Average Effect of Graphene on Natural Frequencies

**Figure 8:**
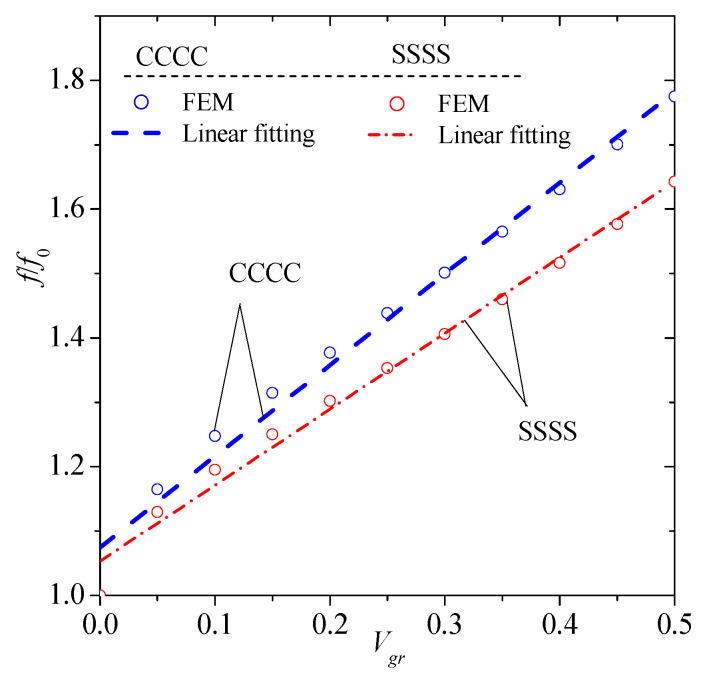
Average effect of graphene on natural frequency of the composite plate of graphene for CCCC, and SSSS boundary conditions.

In order to estimate the average effect of graphene on the natural frequencies of the composite plate, the mean value of the normalized frequency is calculated for every graphene volume fraction considering all modes shape of vibration. Figure 8 depicts the average effect of graphene concerning both CCCC and SSSS boundary conditions. It is obvious that the effect of graphene is greater in CCCC than SSSS boundary conditions. For a rough estimation of the effect for *V_gr_* greater than zero, a linear function can be used, i.e.,

(18) $$\frac{f}{f_{0}}=cV_{gr}+d$$

where *c* is the slope, and *d* is the intercept. In the CCCC case, *c* = 1.4164 and *d* = 1.0745 with R^2^ = 0.9848. In the SSSS case, *c* = 1.1797 and *d* = 1.0533 with R^2^ = 0.9885.

## 4. Conclusions

A multi-scale procedure has been applied in the investigation of the vibration behavior of laminated composite plates reinforced by carbon fibers and graphene. According to analysis results, the following important general conclusions have been drawn:

- The presence of graphene enhanced considerably the orthotropic mechanical properties of the polymer composite lamina;
- The presence of graphene into the composite does not influence the shape of the vibration modes, however it affects their sequence;
- The presence of graphene into the composite increase significantly natural frequencies of the structure;
- The presence of graphene into the composite increase slightly more the normalized frequency of higher order than basic modes;
- The presence of graphene into the composite increase more natural frequencies of CCCC than SSSS boundary condition.

The dispersion of graphene particles in a polymer matrix is a big challenge, and there exists a practical restriction on the addition of graphene above a certain level (maximum of 10 wt.%), owing to the development of agglomerates [39]. Here, we considered a uniform distribution of the graphene in the polymer matrix concerning low- and high-volume fractions. Therefore, the present approach may be used as a design tool for low-volume fractions and be able to reveal the optimum vibrational performance for higher ones.

Future work concerns the attempt of introduction of a correction in the present computational approach considering the non-uniform distribution of nanoparticles in the polymer matrix.

## Figures and Tables

**Table 1:** Fitting parameters of Equation (1) for the prediction of Young’s modulus variation in TPa.

Direction	$P_{0}$	$a_{01}$	$b_{01}$	$b_{02}$	$c_{02}$	$a_{1}$	$b_{1}$	$a_{2}$	$b_{2}$	$c_{2}$
Zig zag	676	13640	−2532	58250	19170	11760	−4.7 × 10^−4^	63.72	85900	17870
Armchair	2.98	3.03	13.55	0.893	−1.197	3.042	19.53	1.1487 × 10^−1^	1.225	−1.634

**Table 2:** Physical properties of components of the composite material.

Carbon Fiber [38]		Polyester Resin [27]		Graphene (Equation (1))	
*Ef*_11_ (MPa)	230,000	*E_m_* (MPa)	3000	*E_gr_* (MPa)	735,000
*Ef*_22_ (MPa)	15,410	*υ_m_*	0.316	*w_gr_* (nm)	10.02
*υf*_23_	0.46	*G_m_* (MPa)	1139.8	*l_gr_* (nm)	9.97
*υf*_12_	0.29	*ρ_m_* (g/cm^3^)	1.20	*ρ_gr_* (g/cm^3^)	2.26
*Gf*_12_ (MPa)	10,040				
*Gf*_23_ (MPa)	5287				
*ρ_f_* (g/cm^3^)	1.80				

**Table 3:** Physical properties of components of the composite material.

*V_gr_*	*E*_1_ (GPa)	*E*_2_ (GPa)	*v*_12_	*v*_23_	*G*_12_ (GPa)	*G*_23_ (GPa)	*ρ_c_* (g/cm^3^)
0.00	139.20	7.799	0.300	0.397	4.924	3.493	1.560
0.05	142.21	13.280	0.300	0.397	8.451	5.732	1.581
0.10	145.47	16.673	0.300	0.397	10.589	7.466	1.602
0.15	149.04	19.828	0.300	0.397	12.552	9.233	1.624
0.20	152.95	23.073	0.300	0.397	14.559	11.124	1.645
0.25	157.25	26.537	0.300	0.397	16.693	13.182	1.666
0.30	162.00	30.305	0.300	0.397	19.010	15.445	1.687
0.35	167.29	34.453	0.300	0.397	21.558	17.953	1.708
0.40	173.20	39.064	0.300	0.397	24.388	20.752	1.730
0.45	179.86	44.237	0.300	0.397	27.562	23.901	1.751
0.50	187.42	50.094	0.300	0.397	31.154	27.473	1.772

## References

[1] Rubino F., Nisticò A., Tucci F., Carlone P. (2020). Marine Application of Fiber Reinforced Composites: A Review. J. Mar. Sci. Eng..

[2] Awad Z.K., Aravinthan T., Zhuge Y., Gonzalez F. (2012). A Review of Optimization Techniques Used in the Design of Fibre Composite Structures for Civil Engineering Applications. Mater. Des..

[3] Ye L., Lu Y., Su Z., Meng G. (2005). Functionalized Composite Structures for New Generation Airframes: A Review. Compos. Sci. Technol..

[4] Narayana K.J., Burela R.G. (2018). A Review of Recent Research on Multifunctional Composite Materials and Structures with Their Applications. Mater. Today-Proc..

[5] Mohammed L., Ansari M.N., Pua G., Jawaid M., Islam M.S. (2015). A Review on Natural Fiber Reinforced Polymer Composite and Its Applications. Int. J. Polym. Sci..

[6] Rajak D.K., Pagar D.D., Menezes P.L., Linul E. (2019). Fiber-Reinforced Polymer Composites: Manufacturing, Properties, and Applications. Polymers.

[7] Georgantzinos S.K. (2017). A New Finite Element for An Efficient Mechanical Analysis of Graphene Structures Using Computer Aided Design/Computer Aided Engineering Techniques. J. Comput. Theor. Nanosci..

[8] Cao G. (2014). Atomistic Studies of Mechanical Properties of Graphene. Polymers.

[9] Georgantzinos S.K., Katsareas D.E., Anifantis N.K. (2011). Graphene Characterization: A Fully Non-Linear Spring-Based Finite Element Prediction. Physica E Low Dimens. Syst. Nanostruct..

[10] Lee J.-H., Park S.-J., Choi J.-W. (2019). Electrical Property of Graphene and Its Application to Electrochemical Biosensing. Nanomaterials.

[11] Georgantzinos S.K., Giannopoulos G.I., Fatsis A., Vlachakis N.V. (2016). Analytical Expressions for Electrostatics of Graphene Structures. Physica E Low Dimens. Syst. Nanostruct..

[12] Prasher R. (2010). Graphene Spreads the Heat. Science.

[13] Falkovsky L.A. (2008). Optical Properties of Graphene. J. Phys. Conf. Ser..

[14] Liu J., Khan U., Coleman J., Fernandez B., Rodriguez P., Naher S., Brabazon D. (2016). Graphene Oxide and Graphene Nanosheet Reinforced Aluminium Matrix Composites: Powder Synthesis and Prepared Composite Characteristics. Mater. Des..

[15] Li G., Xiong B. (2017). Effects of Graphene Content on Microstructures and Tensile Property of Graphene-Nanosheets/Aluminum Composites. J. Alloys Compd..

[16] Abbasi H., Antunes M., Velasco J.I. (2018). Effects of Carbon Nanotubes/Graphene Nanoplatelets Hybrid Systems on the Structure and Properties of Polyetherimide-Based Foams. Polymers.

[17] Irez A.B., Bayraktar E., Miskioglu I. (2020). Fracture Toughness Analysis of Epoxy-Recycled Rubber-Based Composite Reinforced with Graphene Nanoplatelets for Structural Applications in Automotive and Aeronautics. Polymers.

[18] Spanos K.N., Georgantzinos S.K., Anifantis N.K. (2015). Mechanical Properties of Graphene Nanocomposites: A Multiscale Finite Element Prediction. Compos. Struct..

[19] Lin F., Xiang Y., Shen H.S. (2017). Temperature Dependent Mechanical Properties of Graphene Reinforced Polymer Nanocomposites–A Molecular Dynamics Simulation. Compos. Part B Eng..

[20] Montazeri A., Rafii-Tabar H. (2011). Multiscale Modeling of Graphene-And Nanotube-Based Reinforced Polymer Nanocomposites. Phys. Lett. A.

[21] Heidarhaei M., Shariati M., Eipakchi H.R. (2019). Analytical Investigation of Interfacial Debonding In Graphene-Reinforced Polymer Nanocomposites with Cohesive Zone Interface. Mech. Adv. Mater. Struct..

[22] Roach C.C., Lu Y.C. (2017). Analytical Modeling of Effect of Interlayer on Effective Moduli of Layered Graphene-Polymer Nanocomposites. J. Mater. Sci. Technol..

[23] Heidarhaei M., Shariati M., Eipakchi H. (2018). Experimental and Analytical Investigations of the Tensile Behavior of Graphene-Reinforced Polymer Nanocomposites. Mech. Adv. Mater. Struct..

[24] Kumar A., Sharma K., Dixit A.R. (2019). A Review of the Mechanical and Thermal Properties of Graphene and Its Hybrid Polymer Nanocomposites for Structural Applications. J. Mater. Sci..

[25] Rafiee M., Nitzsche F., Laliberte J., Thibault J., Labrosse M.R. (2019). Simultaneous Reinforcement of Matrix and Fibers for Enhancement of Mechanical Properties of Graphene-Modified Laminated Composites. Polym. Compos..

[26] Kostagiannakopoulou C., Loutas T.H., Sotiriadis G., Markou A., Kostopoulos V. (2015). On the Interlaminar Fracture Toughness of Carbon Fiber Composites Enhanced with Graphene Nano-Species. Compos. Sci. Technol..

[27] Taş H., Soykok I.F. (2019). Effects of Carbon Nanotube Inclusion into the Carbon Fiber Reinforced Laminated Composites on Flexural Stiffness: A Numerical and Theoretical Study. Compos. Part B Eng..

[28] Guo H., Cao S., Yang T., Chen Y. (2018). Vibration of laminated composite quadrilateral plates reinforced with graphene nanoplatelets using the element-free IMLS-Ritz method. Int. J. Mech. Sci..

[29] Shen H.S., Xiang Y., Lin F. (2017). Nonlinear vibration of functionally graded graphene-reinforced composite laminated plates in thermal environments. Comput. Methods Appl. Mech. Eng..

[30] Shen H.S., Xiang Y., Fan Y., Hui D. (2018). Nonlinear vibration of functionally graded graphene-reinforced composite laminated cylindrical panels resting on elastic foundations in thermal environments. Compos. Part B Eng..

[31] Shen H.S., Xiang Y., Fan Y. (2017). Nonlinear vibration of functionally graded graphene-reinforced composite laminated cylindrical shells in thermal environments. Compos. Struct..

[32] Wang M., Xu Y.G., Qiao P., Li Z.M. (2019). A two-dimensional elasticity model for bending and free vibration analysis of laminated graphene-reinforced composite beams. Compos. Struct..

[33] Giannopoulos G.I., Liosatos I.A., Moukanidis A.K. (2011). Parametric Study of Elastic Mechanical Properties of Graphene Nanoribbons by a New Structural Mechanics Approach. Physica E Low Dimens. Syst. Nanostruct..

[34] Georgantzinos S.K., Markolefas S.I., Mavrommatis S.A., Stamoulis K.P. (2020). Finite element modelling of carbon fiber-carbon nanostructure-polymer hybrid composite structures. MATEC Web Conf..

[35] Gibson R. (2016). Mechanics of Composite Materials.

[36] Affdl J.H., Kardos J.L. (1976). The Halpin-Tsai equations: A review. Polym. Eng. Sci..

[37] Hibbitt H.D., Karlsson B.I., Sorensen P. (2006). ABAQUS Theory Manual, Version 6.

[38] Kara Y., Akbulut H. (2017). Mechanical behavior of helical springs made of carbon nanotube additive epoxy composite reinforced with carbon fiber. J. Fac. Eng. Archit. Gaz..

[39] Mohan V.B., Lau K.T., Hui D., Bhattacharyya D. (2018). Graphene-based materials and their composites: A review on production, applications and product limitations. Compos. Part B Eng..

